# Silicon Microcantilever Sensors to Detect the Reversible Conformational Change of a Molecular Switch, Spiropyan

**DOI:** 10.3390/s20030854

**Published:** 2020-02-06

**Authors:** Catherine Grogan, George Amarandei, Shauna Lawless, Fran Pedreschi, Fiona Lyng, Fernando Benito-Lopez, Roberto Raiteri, Larisa Florea

**Affiliations:** 1 School of Physics & Clinical & Optometric Sciences, Technological University of Dublin, Kevin Street, D08NF82 Dublin, Ireland; catherine.grogan@tudublin.ie (C.G.); george.amarandei@tudublin.ie (G.A.); fran.pedreschi@tudublin.ie (F.P.); fiona.lyng@tudublin.ie (F.L.); 2 Insight Centre for Data Analytics, National Centre for Sensor Research, Dublin City University, 9 Dublin, Ireland; shauna.lawless27@mail.dcu.ie; 3 FOCAS Institute, Technological University Dublin, Camden Row, 8 Dublin, Ireland; 4 Analytical Microsystems & Materials for Lab-on-a-Chip Group (AMMa-LOAC), Microfluidics Cluster UPV/EHU, Analytical Chemistry Department, University of the Basque Country UPV/EHU, 01006 Vitoria-Gasteiz, Spain; fernando.benito@ehu.eus; 5 Department of Informatics, Bioengineering, Robotics and System Engineering, University of Genova, 16145 Genova, Italy; roberto.raiteri@unige.it; 6 School of Chemistry & AMBER, the SFI Research Centre for Advanced Materials and BioEngineering Research, Trinity College Dublin, the University of Dublin, College Green, 2 Dublin, Ireland

**Keywords:** microcantilever sensor, spiropyran, molecular switch, self-assembled monolayers

## Abstract

The high sensitivity of silicon microcantilever sensors has expanded their use in areas ranging from gas sensing to bio-medical applications. Photochromic molecules also represent promising candidates for a large variety of sensing applications. In this work, the operating principles of these two sensing methods are combined in order to detect the reversible conformational change of a molecular switch, spiropyran. Thus, arrays of silicon microcantilever sensors were functionalized with spiropyran on the gold covered side and used as test microcantilevers. The microcantilever deflection response was observed, in five sequential cycles, as the transition from the spiropyran (SP) (CLOSED) to the merocyanine (MC) (OPEN) state and vice-versa when induced by UV and white light LED sources, respectively, proving the reversibility capabilities of this type of sensor. The microcantilever deflection direction was observed to be in one direction when changing to the MC state and in the opposite direction when changing back to the SP state. A tensile stress was induced in the microcantilever when the SP to MC transition took place, while a compressive stress was observed for the reverse transition. These different type of stresses are believed to be related to the spatial conformational changes induced in the photochromic molecule upon photo-isomerisation.

## 1. Introduction

Silicon microcantilever-based sensors have generated great interest in the last decades due to their high sensitivity and their ability to work as label-free sensors capable of detecting numerous target analytes [1,2,3,4,5]. Early work into microcantilever-based sensors began with the investigations of silicon microcantilevers, typically used with atomic force microscopes, as sensor transducers in their own right [6,7,8]. More recently, microcantilever sensors have proved their sensing abilities in various new areas ranging from gas, humidity and thermal sensing to novel applications in microbiology, genomics and cancer detection [9,10,11,12,13,14,15]. Innovative microcantilever coatings include polymer brushes-based on phenylboronic acid which have been used to detect glucose binding events [15] and graphene oxide (GO) thin films for high-sensitivity humidity sensing [16]. Other examples involve the use of microcantilever sensors for detection of various diseases through, for example, antibody-antigen interactions for the detection of disease-related C-reactive proteins or for the screening of heart-related diseases by using cardiomyocytes functionalized microcantilevers [17,18,19]. Different fabrication methods for microcantilevers as well as the range of available modification methods in the substrate and sensing layer allow for the realization of different types of cantilever-based sensors [20,21]. An improvement in microcantilevers sensor efficiency of the n-type over p-type silicon cantilevers was demonstrated, and this effect was explained by their greater piezoresistive coefficient [20]. Examples include micromachined silicon cantilever paddle sensors which can be used in high flow rate gas sensing [20,21], and resonant cantilevers for pressure sensing [22].

Spiropyrans (SP) are one of the most popular families of photochromic molecules [23,24]. Upon irradiation with UV light, the orthogonal SP isomer converts to the planar merocyanine (MC) form due to the photo-cleavage of the C_spiro_-O bond. The MC isomer shows a strong absorption band in the visible region due to its conjugation. When the MC is exposed to visible light, the structure returns to the SP form. The two isomers (SP vs. MC) have various different properties, including molecular conformations (orthogonal vs. planar), absorption spectra, charge (neutral vs. zwitterion), electric dipole moment (range of ~4–6 D vs. range of ~14–18 D) [25,26,27] and structural differences, whereby SP occupies less volume than MC [28,29,30]. Means of identifying this conformation change commonly include nuclear magnetic resonance (NMR) and optical methods such as UV/Vis, fluorescence, luminescence and optically detected magnetic resonance (ODMR) spectroscopic techniques. The significantly different physico-chemical properties between SP and MC allow for the utilization of SP in a wide variety of applications, such as switchable photo-induced polarity sensors [31], volume change actuators [32,33,34] and wettability modulators [35], as well as for photo-control of binding/release of ions [36,37,38,39], cell adhesion [40] and membrane permeability [41]. Moreover, these molecules can be used to tune surface morphology [33], mechanical properties [42,43] and surface stress [24,44]. Therefore, the combination of microcantilever sensors and SP molecules could generate new types of sensors that synergistically combine the molecular sensing and binding capabilities of the SP-MC pair within a flexible and versatile sensing technology such as the microcantilevers sensors. There is an increased interest in attaching molecular switches, such as SP, to thin films, as they allow for the conversion of molecular response to control, for example, electronic [45], optical [45], wettability [35] and chemosensing properties of materials [45].

Previous work by this group focused on demonstrating the capability of silicon microcantilever sensors to detect the unidirectional conformation change from SP to MC, in the case of Si-bonded self-assembled monolayers (SAMs) and polymeric brushes as a proof of concept [44]. Although successful, several drawbacks such as the inhomogeneity of the generated layer and the tedious coating protocol (e.g., polymer brushes) prevented this technology from being further investigated. In the present work, the gold-coated side of similar silicon microcantilevers has been functionalized with SP-dithiolane SAMs. The limitations of the previous published work were overcome since functionalizing the gold side of the microcantilever, rather than the silicon side, substantially increased the uniformity of the coverage, generating a closely packed configuration of the SP coating when utilizing thiol-terminated SAMs on the microcantilever surface. The dithiolane SP will spontaneously bind to the gold-coated surface of the microcantilever via a strong Au-S interaction ensuring a straightforward surface functionalization. This new system allowed for the detection of the reversible isomerization process between SP and MC over five sequential cycles. This work demonstrates that microcantilevers are able to sense the reversible conformational change of the molecular switch, SP, when functionalized on the gold surface of the microcantilever. The microcantilever deflection provides quantitative information of the stress induced by the conversion between the two isomers of the SP molecules when actuated with light of different wavelengths. This opens the possibility of microcantilever sensor technology with SP sensing capabilities.

## 2. Materials and Methods

### 2.1. Materials

*N, N*’-dicyclohexylcarbodiimide (purity ≥ 99.0%) (DCC), 4₋*N, N*-dimethylaminopyridine (purity ≥ 99.0%) (DMAP), *L*-thioctic acid (purity ≥ 98.0%), dichloromethane (DCM), hexane, ethyl acetate and ethanol were all purchased from Sigma Aldrich (Ireland), and were used as received.

Microcantilever arrays were fabricated from single crystal silicon and are 756 μm long, 150 μm wide, and 1 μm thick (Micromotive GmbH, Germany). Scanning electron microscopy images of the microcantilevers were taken using the Carl Zeiss EVOLS 15 at an accelerating voltage of 10 kV.

### 2.2. Spiropyran Dithiolane Derivative Synthesis

**Figure 1:**
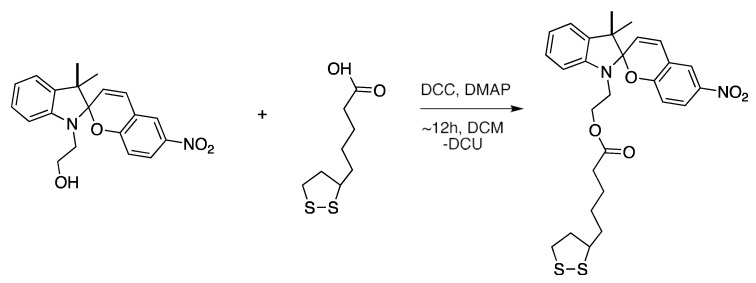
Synthesis of 2-(3′,3′-dimethyl-6-nitrospiro[chromene-2,2′-indolin]-1′yl) -5-(1,2-dithiolan-3-yl)pentanoate from 2-(3′,3′-dimethyl-6-nitrospiro[chromene-2,2′-indolin]-1′-yl) ethanol and L-thioctic acid in the presence of *N*’-dicyclohexylcarbodiimide (DCC) and 4-*N, N*-dimethylaminopyridine (DMAP).

The SP derivative used for the photochromic SAMs, 2-(3′,3′-dimethyl-6-nitrospiro [chromene-2,2′-indolin]-1′yl)-5-(1,2-dithiolan-3-yl)pentanoate (SP-dithiolane), was synthesized from 2-(3′,3′-dimethyl-6-nitrosoiro[chromene-2,2′-indolin]-1′-yl)ethanol (Tokyo Chemicals Industry, United Kingdom) and L-thioctic acid, as described by Ivashenko et al. [46], see Figure 1. The by-product, dicyclohexylurea (DCU) was removed through vacuum filtration, followed by rotary evaporation, in order to remove the DCM solvent. Purification of the product was performed by column chromatography over silica gel, using 1:5:1 hexane:DCM:ethyl acetate as the mobile phase. The isolated product was characterized by ^1^H-NMR and ^13^C-NMR on a Bruker Avance Ultrashield 400 MHz NMR in deuterated chloroform (CDCl_3_) (Figure S1).

### 2.3. UV-Vis Studies

UV-Vis solution studies were performed on a SP-dithiolane 10^−5^ M solution in ethanol using a Varian Carey 50 probe spectrophotometer (Figure S2). Prior to the collection of the MC₋dithiolane spectrum, the solution was irradiated with UV light for 1 min using a CL-1000 Ultra Violet Crosslinker chamber. The kinetics of the SP/MC switching were also investigated in solution (10^−4^ M in ethanol) using the UV/Vis Varian Carey 50 probe spectrophotometer. The absorbance at λ_max_ 545 nm was monitored each second over 1 h (Figure S3). The solution, which was placed in a quartz cuvette, was subjected to UV light until the values stabilized. Following this, the solution was exposed to white light irradiation (Dolan-Jenner-Industries Fiber-Lite LMI light emitting diode (LED lamp)). The experiment was carried out over five cycles. The first-order rate constant for the ring opening (SP to MC) and ring closing (MC to SP) processes were calculated using Equations S1–S2 (ESI, Figures S4 and S5).

### 2.4. Microcantilever Functionalization

Test microcantilevers were functionalized with SP-dithiolane SAMs as per the procedure described by Ivashenko et al. [46]. For this purpose, the microcantilevers were immersed in a 10^−4^ M SP-dithiolane solution in DCM overnight, and then washed with DCM and dried under a gentle stream of nitrogen gas. Each microcantilever array was composed of four wells of microcantilevers (Figure S6A), where the first well was left unfunctionalized (4 reference microcantilevers) while wells 2–4 were functionalized (12 test microcantilevers). This was achieved by having the meniscus of the SP-dithiolane solution below the first well (ESI, Figure S6B).

### 2.5. Microcantilever Deflection Measurements

**Figure 2:**
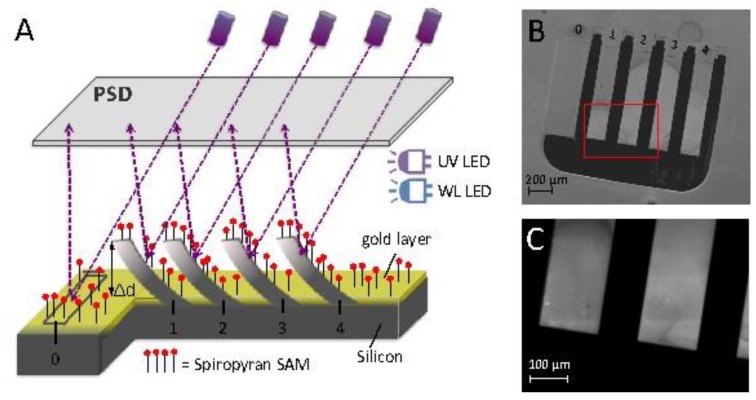
(**A**) Schematic representation of the optical deflection detection method (based on a VCSEL array as the light source and a position-sensitive device (PSD) as detector) for one well of the array comprising of: 0₋reference mirror; 1–4₋SP self-assembled monolayer (SAM) functionalized microcantilevers; The UV LED and white light (WL) LED used for the SP/MC switching are also shown. (**B**) SEM image of one individual well composed of 4 microcantilevers and one reference mirror; (**C**) SEM detail of microcantilevers.

Microcantilever deflection measurements were carried out using the Protiveris system, which utilizes the static mode of microcantilever operation [47]. The deflection of 16 microcantilevers can be measured simultaneously with this system (Figure 2). The position-sensitive detector (PSD) has a 20 × 20 mm^2^ sensing area, and allows measurement of microcantilever bending down to 0.1 nm [47]. The microcantilever array contains four individual cells (Figure 2A, each of them composed of four free microcantilevers and one fixed mirror, as seen in Figure 2A,B The fixed mirror is in place to give information on perturbations/drifts of the optical system and not from the deflection of cantilevers. The deflection of each individual microcantilever was measured after achieving an optical alignment of the laser sources on the back of the microcantilever array. In this work, post SP functionalization, all the cantilevers were deflected upwards with respect to the reference mirror. This caused a difference in the position of the reflected spots that prevented getting the maximum laser reflection from the back of the free cantilevers and the fixed mirror, simultaneously. As a direct consequence, it was not possible to monitor the signal from the fixed mirrors in these experiments. A method to overcome this limitation is difficult to implement due to the functionalization of the cantilevers. Therefore, further studies will be needed in order to overcome this limitation. The microcantilevers were maintained at the same environmental temperature of 26 °C throughout the experiments. The cartridge holder is contained in an environmental chamber blocking outside light. The cartridge is thermally insulated from the rest of the device by a thick Teflon sheet and it can be heated using a resistor embedded in the metal body of the cartridge itself. The temperature can be controlled with 0.1 °C precision using an external temperature controller, as described in [47]. A white light emitting diode (LED) and a UV LED were placed directly over the microcantilever holder and inside the chamber enclosing the microcantilever holder. The white or UV LED were turned on or off at the required times. LED sources were used in order to minimize possible heating effects of the microcantilevers that could lead to associated thermal drifts. Data normalization and baseline subtraction methods were utilized as described by Hegner et al. [48,49].

### 2.6. Detection of Surface Stress

A difference in surface stress between the two sides of a microcantilever induces a deflection of the microcantilever. This change in surface stress and corresponding deflection can be calculated using a version of Stoney’s formula, Equation (1) [2],

(1) $$\Delta z=\;\frac{3\left(1-v\right)L^{2}}{\hat{E}t^{2}}\sigma$$

where L is the length (756 µm), v is the Poisson’s ratio (0.22), $\hat{E}$ is the Young’s Modulus (1.65 × 10^11^ Pa), t is the thickness (1µm) of the microcantilever and σ is the surface stress generated. The surface stress induced on the microcantilever surface, as a result of the SP/MC conversion, can be calculated by using the average differential deflection and Equation (1).

## 3. Results and Discussion

### 3.1. Photo-Induced Microcantilever Deflections

Previous work tested the response of the silicon microcantilevers functionalized with SP on the silicon side of the microcantilevers, monitoring only the change in state from SP to MC and without using baseline subtraction methods [44]. This earlier work showed an upward deflection response of the functionalized microcantilever on exposure to UV due to the SP changing state to the MC form. In the present work, the microcantilevers having a SP-dithiolane SAM coating on the gold side (test microcantilevers) showed a downward deflection upon similar exposure to UV light.

**Figure 3:**
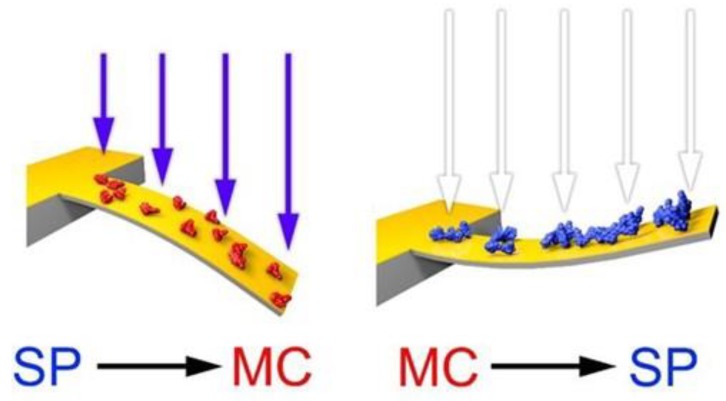
Schematic representation of deflection of SP functionalized cantilever when changing state form the SP to the MC form and vice versa.

**Figure 4:**
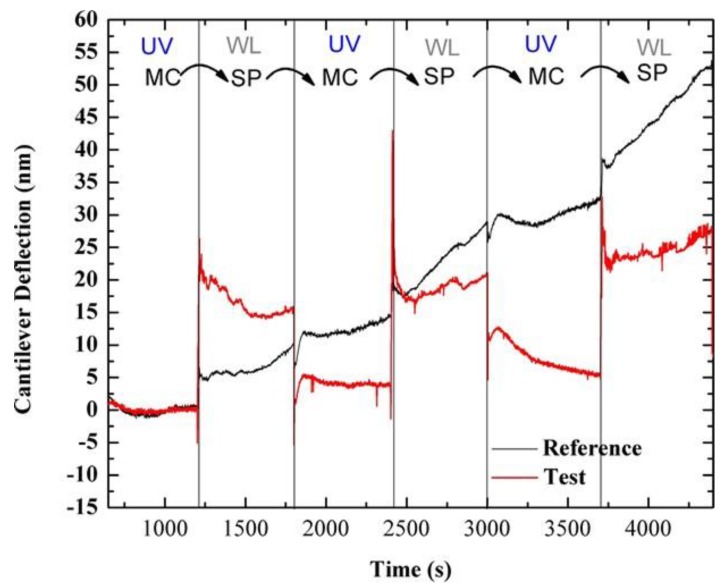
Typical average deflection response of the test (red) and reference (black) microcantilevers during 5 cycles of alternating UV and white light illumination.

The different type of stress observed in these experiments, between the SP and MC state, can be explained if one considers the spatial arrangements of the photochromic molecules, where SP occupies less volume than MC [23,50]. As the transition SP–MC takes place under UV irradiation, the MC isomer tends to occupy a larger volume and this can be accommodated by the microcantilever only through a change in its curvature in order to increase its surface area. This leads to the occurrence of a tensile stress and down deflection (schematic representation Figure 3, and deflection data shown in Figure 4). On the other hand, the MC to SP transition implies a reduction in the surface area covered by the photochromic molecule and a minimization in its free energy. Consequently, the microcantilever tends to reduce its area, i.e., bending in the opposite direction (Figure 4 and the schematic representation in Figure 3). The magnitude of the stress values presented will depend on the surface density of the photochromic molecules [35,44].

Tests were also carried out to determine the ability of SP-functionalized microcantilevers to detect the reversibility of the SP–MC transition. To this end, microcantilever response was measured when exposed to successive illumination using UV and white light LEDs. Figure 4 shows a typical average deflection response of the test and reference microcantilevers from one array on exposure to UV and white LEDs over five sequential switching cycles (optimum alignment microcantilevers signals used, n = 2 reference, n = 3 test). Despite the drift in the deflection of the reference microcantilever that can be mainly ascribed to thermal fluctuations, larger deflection responses can be clearly observed in test microcantilevers on exposure to the UV and white light LEDs; thus demonstrating the ability of silicon microcantilever sensors to detect the reversible change of SP between the SP and MC state when considering the differential deflection (test minus reference). The fact that the deflection of the test microcantilevers in both the SP and MC states appears more constant than the reference microcantilevers is due to the fact that the surface stress induced by the SP or MC conformations is relatively constant. The fast response time is the result of the monolayer coverage allowing for fast switching of the SP coating. Larger equilibrium time scales are typically observed in the case of polymeric coatings [15] or microcantilever deflections that are a result of binding events [51,52].

**Figure 5:**
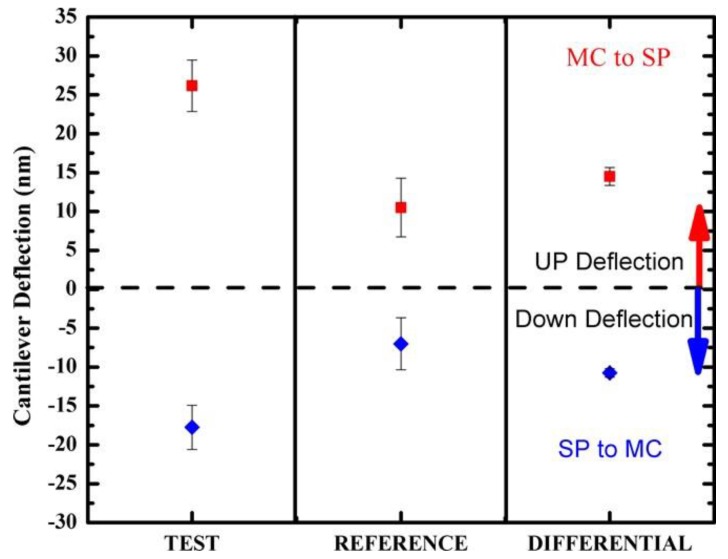
Average test, reference and differential microcantilever deflection response on exposure to UV and white light LEDs over five switching cycles as extracted from Figure 4.

The mean and standard error of the microcantilever deflection for the test, the unfunctionalized reference and the differential deflections were determined by measuring responses to UV or white light LEDs over five switching cycles. The mean linear deflections of the test and reference microcantilevers were calculated using data normalization and baseline subtraction (see [49,51] for more details) and are presented in Figure 5. The differential deflection response of the test minus reference microcantilevers during 5 cycles of alternating UV and white light illumination are presented in Figure S7. It can be seen that the MC–SP transition leads to a mean differential microcantilever deflection of 15 ± 1 nm upwards, while the SP–MC transition leads to a mean differential microcantilever of 10.8 ± 0.6 nm, downwards; see Figure 5. These deflection changes, in the tens of nm range, are due to molecular conformational changes. Microcantilever deflections presented in Figure 4 are showing a difference in the direction of deflection and therefore type of stress induced when switching between the SP and MC states. These results indicate a different spatial layout of the SP molecules when either in the SP or MC states on the microcantilever surface, which concurs with other findings on the structural differences of the SP and MC [28,29,30,42].

### 3.2. Photo-Induced Surface Stress

The surface stress induced on the microcantilever surface as a result of the SP/MC conversion was calculated and is presented in Table 1.

Further studies are needed in order to understand the relationship between the aerial surface coverage, the spatial distribution of the photochromic molecules and the induced stress. Similar microcantilever deflections and surface stress changes have been observed in other studies where gold coated microcantilevers were used for the detection of a gene mutation linked to skin cancer [10] or detection of Kanamycin [52]. However, a quantitative comparison to existing literature cannot be made due to the difference in the microcantilever sensing systems and in the coating on the microcantilever surface. Previous investigations exploiting microcantilever sensors for detection of molecular conformation changes most commonly firstly implied a ligand–receptor association or dissociation event (accompanied by a mass alteration) that resulted in a molecular change in the microcantilever coating. As an example, the conformation change of the protein bacteriorhodopsin, immobilized on the microcantilever surface, has been detected by measuring the microcantilever deflection response to the injection of hydroxylamine, which induces conformation changes in this protein due to ligand receptor dissociation [49]. Another study has demonstrated the ability to monitor real-time conformational changes of enzymes immobilized on a microcantilever surface as a result of binding with their ligands [53].

At present, elucidating the origin and the magnitude of the stress induced by the SP–MC transitions (and reflected by the cantilever deflection) still needs further investigations. Thus, while it is clear that different spatial configuration are occupied by the SP and MC state [46,54], further factors might also contribute (by enhancing or hindering) to the detected deflection. Thus, the presence of absorption/emission/dissipation phenomena in the functionalized cantilever can also play a role, and they can mainly contribute to the presence of the thermal drift on the bending. The role of the thermal drift appears to be mainly in hindering the effect of the bending, but further studies will be needed in order to elucidate these aspects. Additionally, improvements in the system to reduce the effects of the thermal drift would be beneficial. Other groups have developed systems with improved temperature isolation and methods to measure the static and dynamic deflections of the microcantilevers to increase the accuracy of the measurements [11,55]. Such improvements are foreseen for the present configuration and will contribute in elucidating the phenomena described in this paper. Even though such sophisticated improvements were not present in the work, the obtained results clearly show that microcantilevers can sense reversible molecular conformation changes of the SP monolayer solely due to isomerization. Moreover, the lack of hysteresis during the light cycling (UV/WL) indicates that the microcantilever is an effective transducer for characterizing this reversible photoswitching process.

## 4. Conclusions

In this study, reversible photo-induced isomerization between the SP and MC states has been detected and evaluated using silicon microcantilever-based sensors. For this, the gold coated side of the microcantilever sensor was functionalized with SP SAMs. Testing five switching cycles of SP to/from MC transitions demonstrated the ability of the microcantilever sensor system to detect the reversible switching between the two states on exposure to white or UV light, which induced a tensile and compressive stress for the SP to MC and MC to SP transitions, respectively. This difference in surface stress can be interpreted in terms of the different surface spatial arrangements of the two isomers.

This work also represents a first step towards the realization of a microcantilever sensor, which can be remotely and selectively configured using UV and white light LEDs. Based on the well-known binding properties of the MC isomer, once in the ‘OPEN’ MC state, the microcantilever could be used to capture and detect cationic species such as divalent metal ions by monitoring microcantilever deflection. Future work in this area will further develop this microcantilever system and will test its ability to detect the presence of metal ions when the SP coating is turned ‘OPEN’ remotely.

## Figures and Tables

**Table 1:** The surface stress induced as a result of SP/MC photo-isomerization.

State Transition	Surface Stress Induced (N m^−1^)	Type of Stress
SP → MC	−1.3 ± 0.1 × 10^−3^	Compressive
MC → SP	+1.8 ± 0.1 × 10^−3^	Tensile

## Supplementary Materials

The following are available online at https://www.mdpi.com/1424-8220/20/3/854/s1, Figure S1: ^1^H NMR of SP-dithiolane; Figure S2: Absorbance spectrum of a 10^−5^ M solution of SP-dithiolane in ethanol, under different illumination conditions; Figure S3: Graph monitoring the absorbance at λ_max_ = 545 nm during UV/Vis irradiation cycles of a SP-dithiolane solution in ethanol; Figure S4: Experimental data and fitted model following the absorbance at 545 nm during the ring opening (SP to MC) process, under UV irradiation, for three consecutive switching processes; Figure S5: Experimental data and fitted model following the absorbance at 545 nm during the ring closing (MC to SP) process, under white light irradiation, for three consecutive switching processes; Figure S6: (A) Photograph of two cantilever arrays having the silicon (top) and the gold (bottom) side facing up; (B) Placement of the cantilever array, during functionalisation, using the SP-dithiolane solution; Figure S7: Typical differential deflection response of the test microcantilever during 5 cycles of alternating UV and white light illumination.
