# Supplementary material for: Silicon Microcantilever Sensors to Detect the Reversible Conformational Change of a Molecular Switch, Spiropyan
Source: Sensors (Basel). 2020 Feb 6;20(3):854. doi: 10.3390/s20030854 (PMC7039217; doi:10.3390/s20030854)
Supplement: Supplementary file 1 [file sensors-20-00854-s001.pdf]

Supplementary Information

Silicon microcantilever sensors to detect the reversible conformational change of a molecular switch, spiropyran

Catherine Grogan,[1] George Amarandei, [1] Shauna Lawless,[2] Fran Pedreschi,[1] Fiona Lyng,[1,3] Fernando Benito-Lopez,[4] Roberto Raiteri[5] and Larisa Florea[6,*]

[1] School of Physics & Clinical & Optometric Sciences, Technological University of Dublin, Kevin Street, D08NF82, Dublin 8, Ireland; catherine.grogan@tudublin.ie (C.G.); george.amarandei@tudublin.ie (G.A.); fran.pedreschi@tudublin.ie (F.P.)
[2] Insight Centre for Data Analytics, National Centre for Sensor Research, Dublin City University, Dublin 9, Ireland
[3] FOCAS Institute, Technological University Dublin, Camden Row, Dublin 8, Ireland; fiona.lyng@tudublin.ie
[4] Analytical Microsystems & Materials for Lab-on-a-Chip Group (AMMa-LOAC), Microfluidics Cluster UPV/EHU, Analytical Chemistry Department, University of the Basque Country UPV/EHU, Vitoria-Gasteiz 01006, Spain; fernando.benito@ehu.eus
[5] Department of Informatics, Bioengineering, Robotics and System Engineering, University of Genova, 16145 Genova, Italy; roberto.raiteri@unige.it
[6] School of Chemistry & AMBER, the SFI Research Centre for Advanced Materials and BioEngineering Research, Trinity College Dublin, the University of Dublin, College Green, Dublin 2, Ireland
* Correspondence: floreal@tcd.ie

Table of Contents:

S.1: H^1 and ^{13}C NMR Analysis of SP-dithiolane

S.2: UV-Vis Analysis of SP-dithiolane in solution

S.3: Cantilever Functionalisation Protocol

S.4: Differential (Test minus Reference) cantilever deflection over 5 cycles of UV and white light exposure.

S.1: ^{1}H and ^{13}C NMR Analysis of SP-dithiolane

^{1}H NMR ($CDCl_3$): δ 1.18 (s, 3H), 1.21 (s, 3H), 1.34 (m, 2H), 1.53 (m, 4H), 1.80 (m, 1H), 2.19 (m, 2H), 2.34 (m, 1H), 3.02 (m, 2H), 3.09 (m, 1H), 3.34 (m, 2H), 4.09 (m, 1H), 4.17 (m, 1H), 5.81 (d, 1H), 6.63 (d, 1H), 6.68 (d, 1H), 6.81 (m, 2H), 7.02 (d, 1H), 7.12 (t, 1H), 7.94 (m, 2H).

^{13}C-NMR ($CDCl_3$): δ 19.85, 24.54, 25.90, 28.74, 31.00, 33.92, 38.50, 40.24, 42.39, 52.85, 56.31, 62.43, 106.48, 106.74, 115.57, 118.42, 119.92, 121.77, 121.85, 122.80, 125.99, 127.85, 128.36, 135.61, 141.03, 146.70, 159.43, 173.31.

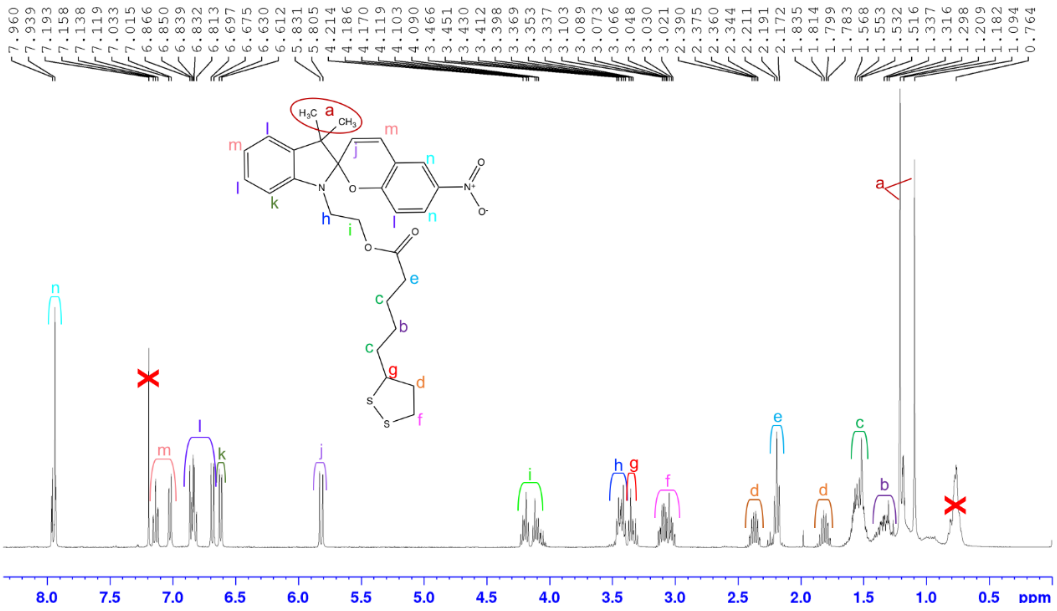

Figure S1. ^{1}H NMR for the spiropyran product, 2-(3’,3’-dimethyl-6-nitrospiro[chromene-2,2’-indolin]-1’yl)-5-(1,2-dithiolan -3-yl)pentanoate; The peak ~ 0.8 ppm indicates the presence of hexane, which was used during the purification stage. The peak ~ 7.2 ppm indicates the presence of $CDCl_3$, which was used as the solvent for ^{1}H NMR analysis.

S.2: UV-Vis Analysis of SP-dithiolane in solution

The absorbance spectra of the SP-dithiolane solution (10^{-5} M in ethanol) was recorded after UV and white light exposure, respectively (Figure S2). The absorption spectrum in Figure S2 shows the difference in absorption of SP-dithiolane after UV light and white light exposure. The spectrum after UV irradiation represents the MC isomer which has a λ_{max} of ~ 545 nm.

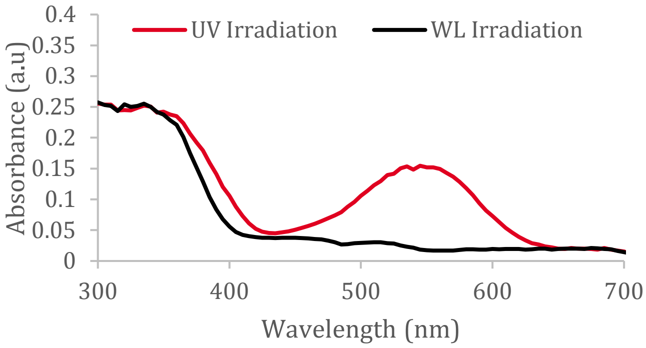

Figure S2. Absorbance spectrum of a 10^{-5} M solution of SP-dithiolane in ethanol, under different illumination conditions.

The reversible switching process between SP/MC was repeated several times. Under UV irradiation, the SP-dithiolane (10^{-5} M solution in ethanol) isomerised to the MC form, causing an increase in the absorbance at λ_{max} ~ 545 nm. Once this absorbance was stabilised, the UV light was removed and white light was introduced. This led to a dramatic drop in the absorbance at λ_{max} 545 nm, indicating the switching of the MC form back to the SP isomer. This process was repeated four times indicating that the photo-induced isomerisation is reversible, happens rapidly, and that the isomers stabilise relatively fast in solution under constant irradiation.

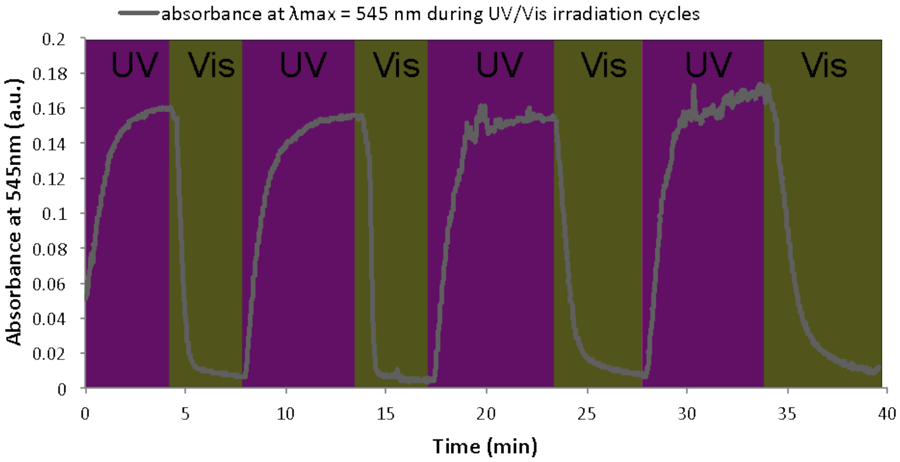

Figure S3. Graph monitoring the absorbance at λ_{max} = 545 nm during UV/Vis irradiation cycles of a SP-dithiolane solution in ethanol. UV irradiation causes the switching of the SP to the MC form, resulting in an increase in the absorbance at λ_{max}; Following this, the UV light is removed and replaced with white light (Vis), causing the switching of the MC back to the SP form. This process is monitored through the decrease in the absorbance at λ_{max}. The cycle was repeated four times.

The rate constant for the ring-opening process was calculated using Eq. S1

$$y = a\left(1 - e^{-(kt)}\right) + b \quad \text{(S1)}$$

where y is the absorbance at λ_{max}, a is the scaling factor, k is the first order rate constant (min^{-1}), t is the time (min), and b is the baseline offset.

The average value for k (rate constant) for the ring-opening process was found to be 1.3 ± 0.1 min^{-1} (n = 3, Figure S4). The graphs show the absorbance at 545 nm increasing over time, before reaching a plateau after ~ 3 min of UV irradiation (Figure S4).

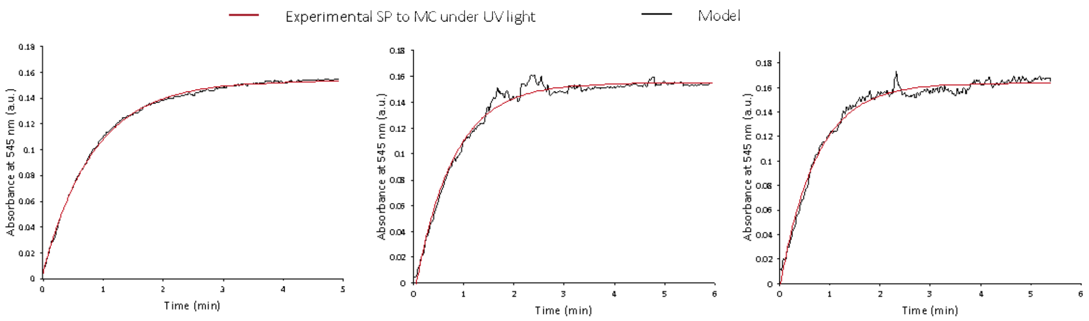

Figure S4. Experimental data and fitted model following the absorbance at 545 nm during the ring opening (SP to MC) process, under UV irradiation, for three consecutive switching processes.

The rate constant for the ring-closing process was calculated using Eq. S2.

$$y = a\left(e^{-(kt)}\right) + b \quad \text{(S2)}$$

Where y is the absorbance at λ_{max}, a is the scaling factor, k is the first order rate constant (min^{-1}), t is the time (min), and b is the baseline offset.

The average value for k (rate constant) for the ring-closing process was found to be 2 ± 1 min^{-1} (n = 3, Figure S5). The MC to SP switching under white light occurs faster compared to the reverse process in the current experimental conditions (solvent, concentration, light sources) and the absorbance reaches a plateau after ~ 2 min of white light irradiation.

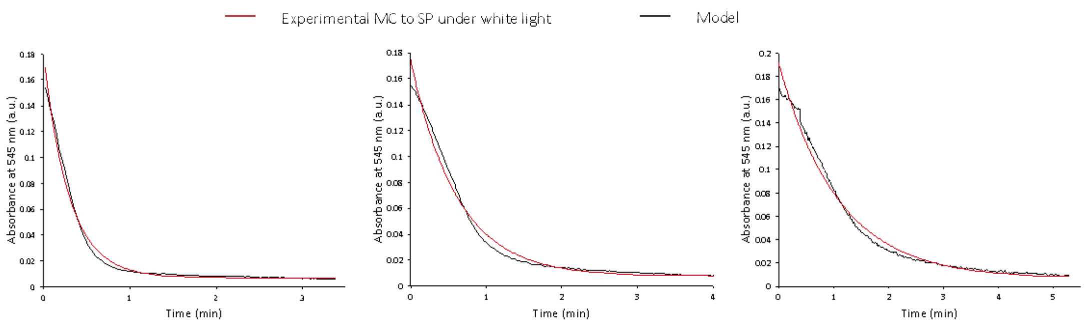

Figure S5. Experimental data and fitted model following the absorbance at 545 nm during the ring closing (MC to SP) process, under white light irradiation, for three consecutive switching processes.

S.3: Cantilever Functionalisation Protocol

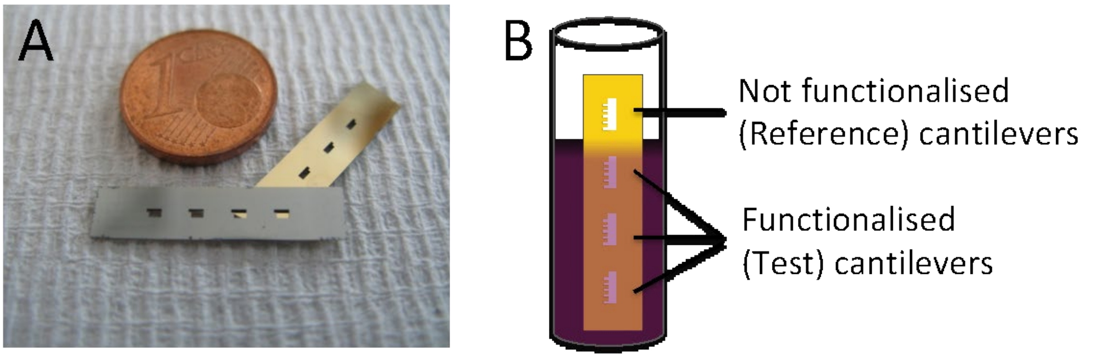

Figure S6. (A) Photograph of two cantilever arrays having the silicon (top) and the gold (bottom) side facing up; (B) Placement of the cantilever array, during functionalisation, using the SP-dithiolane solution (coloured in purple); The vial was closed and no solvent evaporation was observed during the functionalisation process.

S.4.: Differential (Test minus Reference) cantilever deflection over 5 cycles of UV exposure.

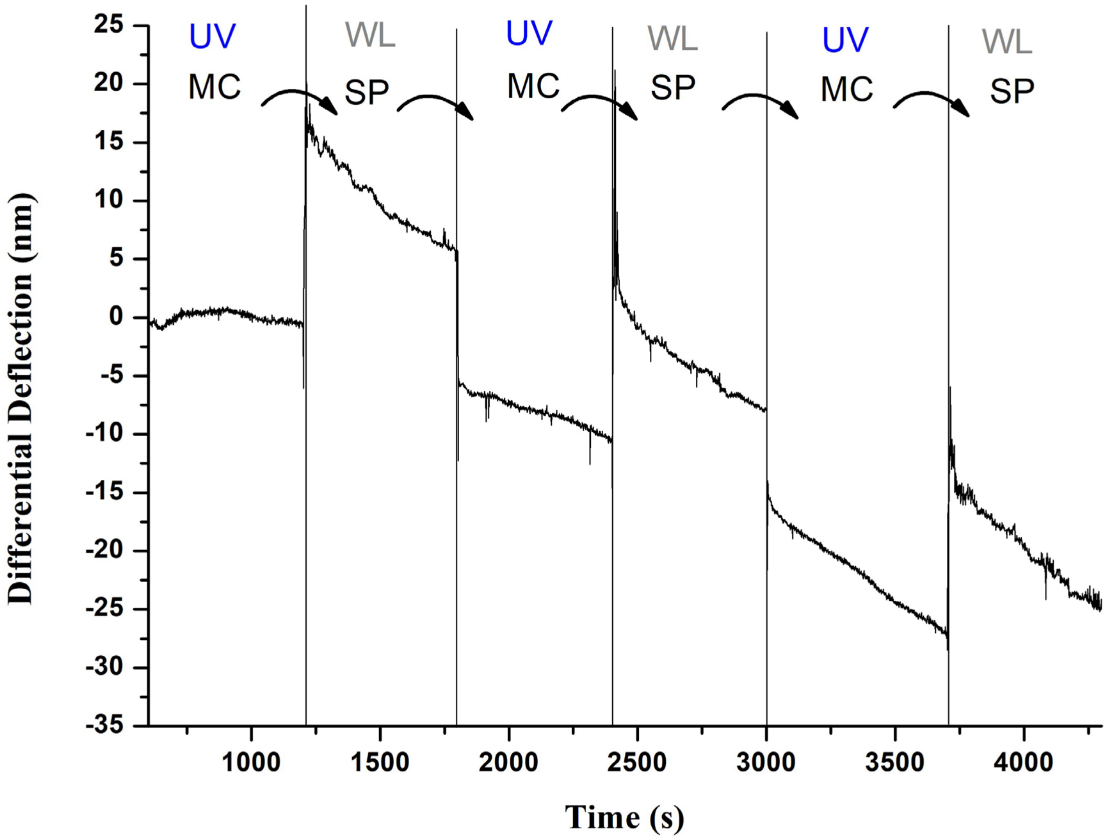

Figure S7. Typical differential deflection response of the test minus reference microcantilevers during 5 cycles of alternating UV and white light illumination.
